# Supplementary material for: HCN1 is a primary HCN Pacemaker Channel in Neurons
Source: Nat Commun. 2026 Apr 23;17:3745. doi: 10.1038/s41467-026-72257-3 (PMC13106851; doi:10.1038/s41467-026-72257-3)
Supplement: Supplementary file 1 — Supplementary Information [file 41467_2026_72257_MOESM1_ESM.pdf]

Supplementary Information

to the manuscript

HCN1 is a primary HCN Pacemaker Channel in Neurons

Uta Enke, Andrea Schweinitz, Debanjan Tewari, Christian Sattler, Ralf Schmauder, Christoph Schmidt-Hieber, Klaus Benndorf

Contents:
7 Supplementary Figures
7 Supplementary Tables

Supplementary Figures

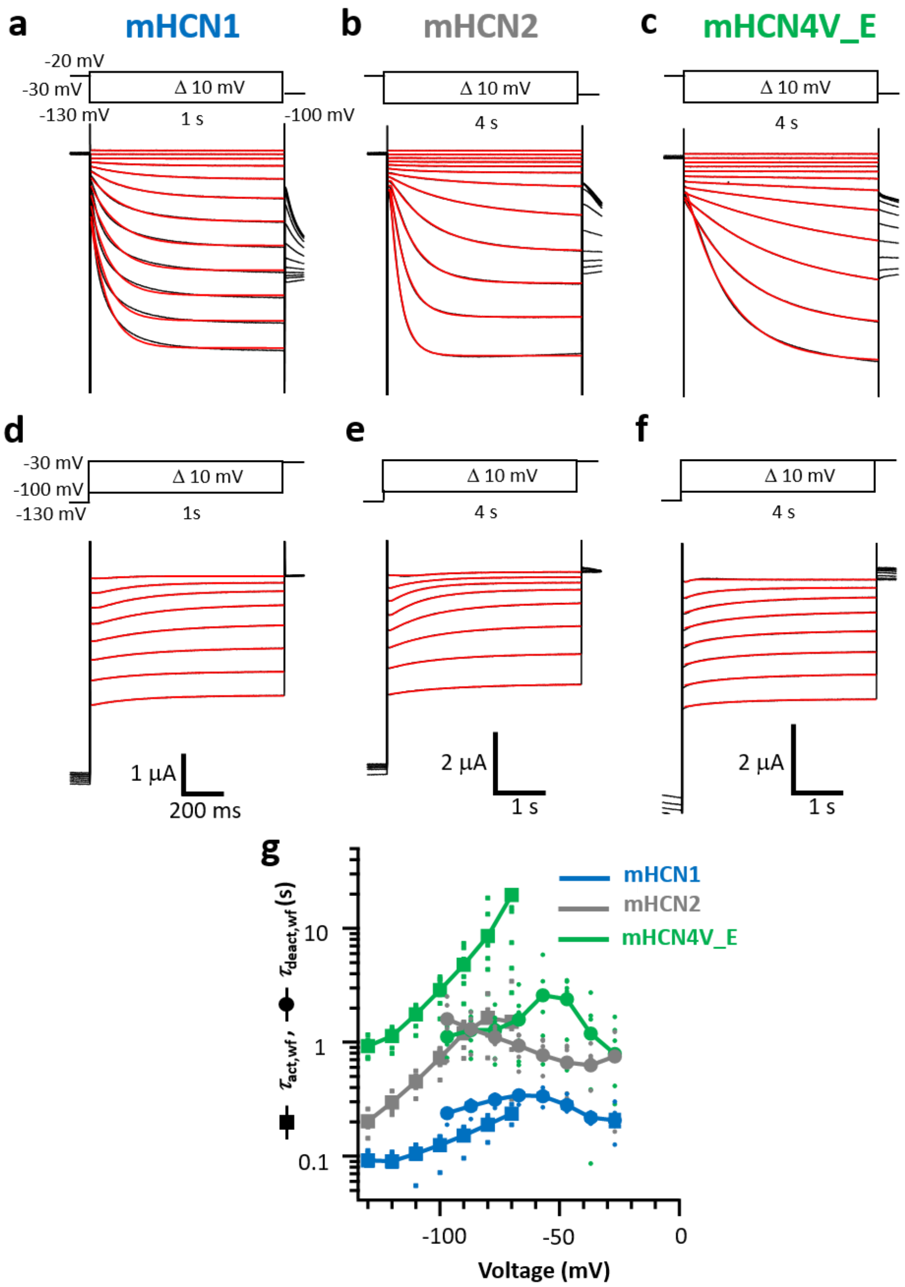

Supplementary Fig. 1 | Voltage dependence of activation and deactivation with conventional protocol by TEVC. The voltage protocols are indicated above the traces. **(a-c)** Families of currents determined with the TEVC technique for mHCN1, mHCN2 and mHCN4V_E channels to determine the kinetics by the fast voltage protocols. Pulsing frequency 0.333 Hz mHCN1, 0.125 mHCN2 and mHCN4V_E. The activation time courses were fitted by an exponential function according to equation (3) yielding the time constant $\tau_{act,wf}$ (red curves). **(d-f)** Respective deactivation time courses. Pulsing frequency 0.333 Hz mHCN1, 0.1 Hz mHCN2 and mHCN4V_E. Deactivation time courses were fitted by an exponential yielding the time constant $\tau_{deact,wf}$ (red curves). **(g)** Time constants for activation ($\tau_{act,wf}$; filled circles) and deactivation ($\tau_{deact,wf}$; filled squares) as function of voltage. Large symbols represent the means and small symbols the individual measurements (n=6).

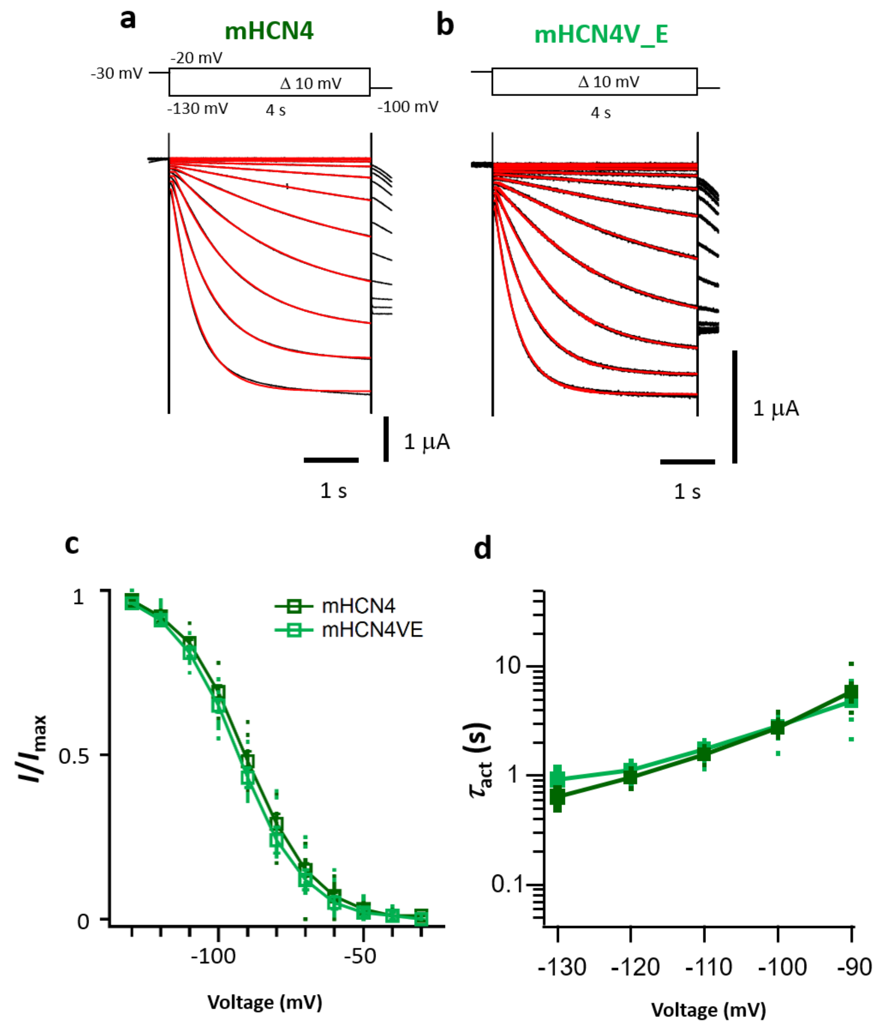

Supplementary Fig. 2 | In TEVC activation of mHCN4V_E and mHCN4 are closely similar. (a,b) Current families through mHCN4V_E and mHCN4 obtained by TEVC. Pulsing frequency 0.067 Hz. **(c)** Steady-state activation. The means of the data points were fitted by equation (2) yielding V_{hw4m}=-87.8 mV and $z\delta_{w4m}$=2.2 for mHCN4V_E (n=7) and V_{hw4}=-90.5 mV and $z\delta_{w4}$=2.3 for mHCN4 (n=8). **(d)** Plot of the corresponding activation time constants τ_{act} as function of voltage. Small symbols show the individual measurements.

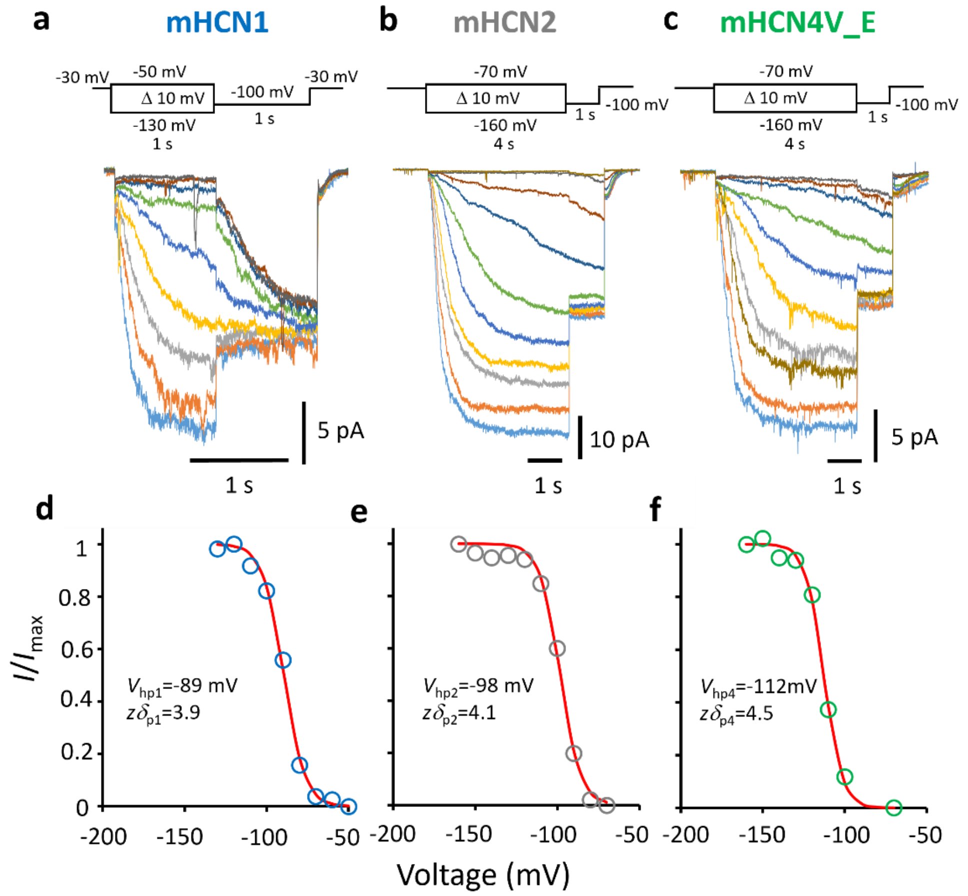

Supplementary Fig. 3 | Steady-state activation of mHCN1, mHCN2 and mHCN4V_E in cell-attached patches. (a-c) Families of current traces. The voltage protocols correspond to those in the TEVC recordings in Supplementary Fig. 1. **(d-f)** Corresponding plots of steady-state activation relationships for the shown recordings. The traces were fitted with equation (2). The parameters are indicated.

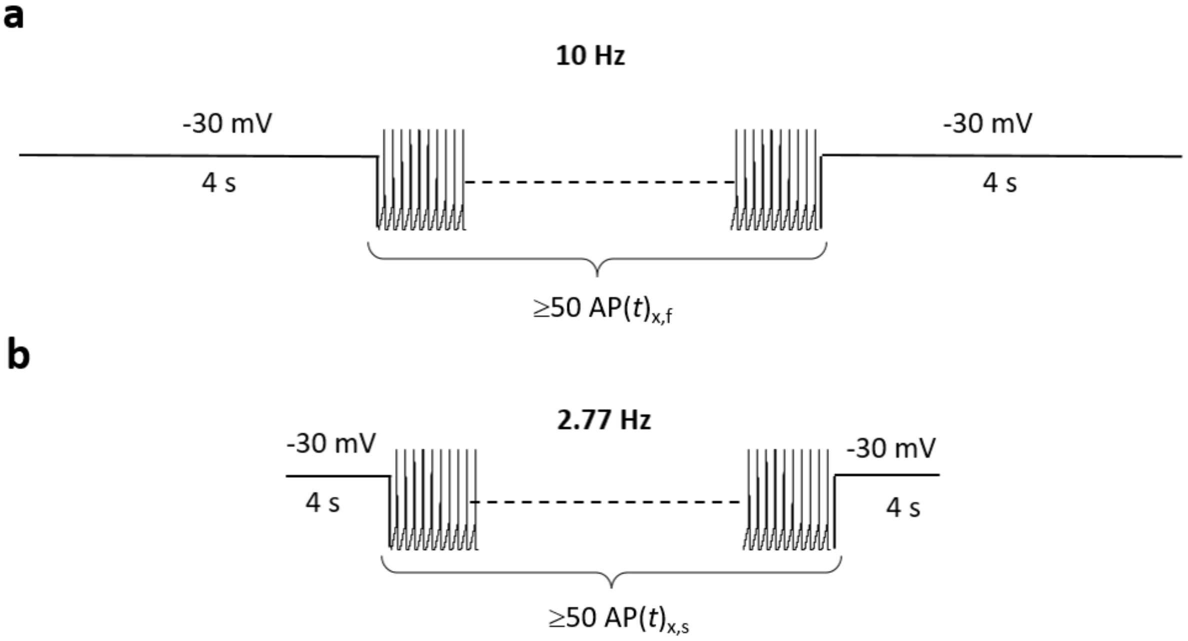

Supplementary Fig. 4 | Action potential trains used as command potentials. (a) Fast protocol: After a 4 s pulse to -30 mV, a train of ≥50 APs at 10 Hz was applied that was followed by a 4 s pulse back to -30 mV. **(b)** Slow protocol: After a 4 s pulse to -30 mV, a train of ≥50 APs at 2.77 Hz was applied that was followed by a 4 s pulse back to -30 mV.

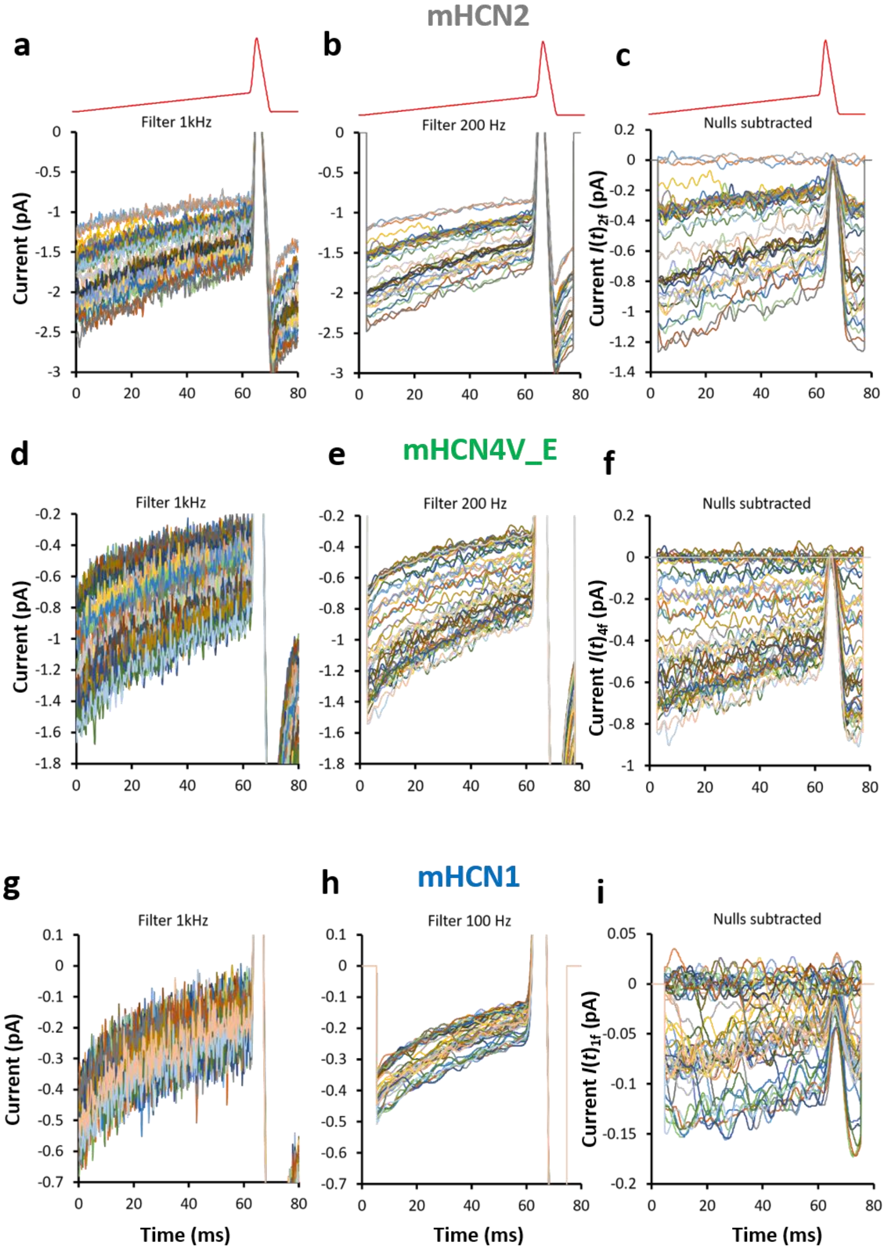

Supplementary Fig. 5 | Pacemaker currents at 10 Hz AP trains. (a-c) mHCN2. The traces correspond to Fig. 3a-d. **(d-f)** mHCN4V_E. The traces correspond to Fig. 3e-h. **(g-i)** mHCN1. The traces correspond to Fig. 3i-l.

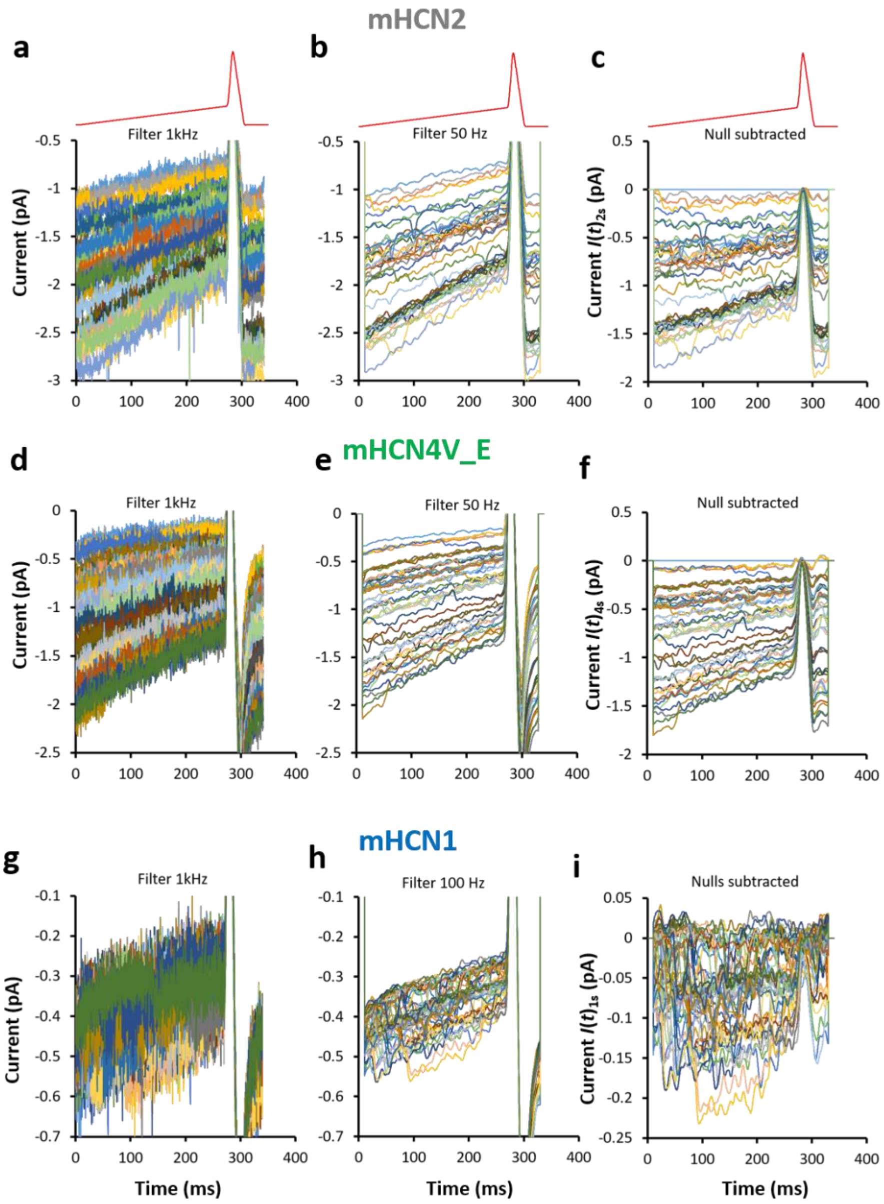

Supplementary Fig. 6 | Pacemaker currents at 2.77 Hz AP trains. (a-c) mHCN2. The traces correspond to Fig. 4a-d. **(d-f)** mHCN4V_E. The traces correspond to Fig. 4e-h. **(g-i)** mHCN1. The traces correspond to Fig. 4i-l.

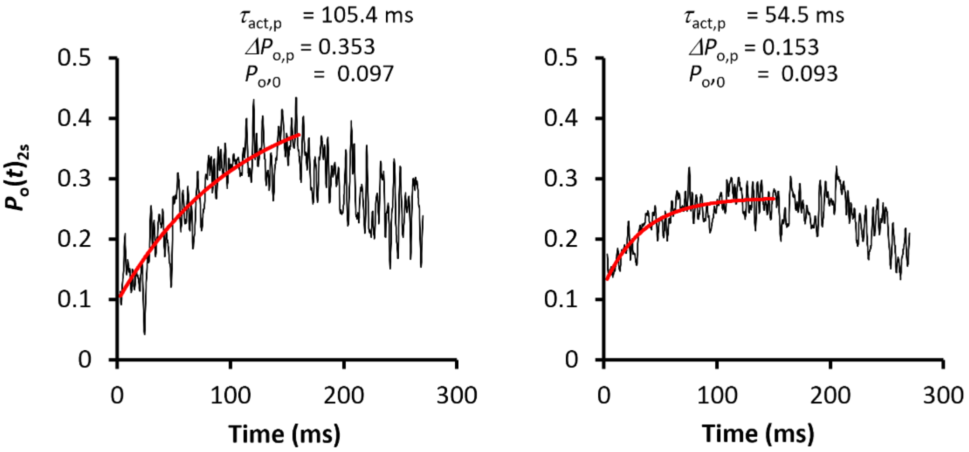

Supplementary Fig. 7 | Two representative time courses of $P_o(t)_{1s}$ of mHCN1 channels at the slow AP frequency of 2.77 Hz. $P_o(t)_{1s}$ shows pronounced activation followed by deactivation during the pacemaker depolarization. The time course was fitted (red curve) in the indicated interval by equation (1) yielding the indicated parameters.

Supplementary Tables

Supplementary Table 1 | Parameters for steady-state activation in TEVC and cell-attached patches. V_{hx} and $z\delta_x$ indicate half maximum activation and the apparent gating charge, respectively, obtained by fitting equation (2). ‘w’ and ‘p’ indicates whole-cell and patch, respectively. ‘x’ indicates the channel type 1,2, 4m, and 4 for mHCN1, mHCN2, mHCN4V_E, and mHCN4, respectively. Asterisks indicate values obtained from our previous report[13].

	TEVC			Cell-attached patch			ΔV_x (mV)
**Channel**	V_{hwx} (mV)	$z\delta_{wx}$	n	V_{hpx} (mV)	$z\delta_{px}$	n	
**mHCN1**	-63±1*	3.1±0.2*	8	-89±3	4.3±0.5	11	-26
**mHCN2**	-79±1*	3.3±0.3*	5	-107±3	5.6±0.4	10	-28
**mHCN4V_E**	-88±2	2.2±0.1	7	-113±2	3.1±0.3	6	-25
**mHCN4**	-91±1*	2.3±0.1*	8				

Supplementary Table 2 | Statistics of the patches used for determining P_o during the pacemaker depolarization for mHCN2 channels. AP frequency 10 Hz.

mHCN2 - AP frequency 10 Hz			
**Patch No.**	**Measurement No.**	**# Traces**	**Po**
**1**	1	45	0.059
**2**	2	49	0.225
**3**	3	179	0.244
	4	48	0.008
**4**	5	169	0.667
	6	50	0.349
	7	50	0.156
**5**	8	49	0.152
**6**	9	129	0.754
	10	50	0.181
	11	47	0.099
**7**	12	116	0.594
	13	42	0.344
	14	40	0.244
**8**	15	147	0.151
**Sum**		**1210**	
**Mean**			**0.282**

Supplementary Table 3 | Statistics of the patches used for determining P_o during the pacemaker depolarization for mHCN4V_E channels. AP frequency 10 Hz.

mHCN4V_E - AP frequency 10 Hz			
**Patch No.**	**Measurement No.**	**# Traces**	**Po**
**1**	1	189	0.388
	2	50	0.106
	3	50	0.041
**2**	1	50	0.142
	2	48	0.149
	3	50	0.169
**3**	1	224	0.347
	2	46	0.118
**4**	1	207	0.234
	2	206	0.182
	3	63	0.335
**5**	1	241	0.494
	2	50	0.118
**6**	1	172	0.313
	2	50	0.081
	3	94	0.382
**Sum**		**1790**	
**Mean**			**0.225**

Supplementary Table 4 | Statistics of the patches used for determining P_o during the pacemaker depolarization for mHCN1 channels. AP frequency 10 Hz.

mHCN1 - AP frequency 10 Hz			
**Patch No.**	**Measurement No.**	**# Traces**	**Po**
**1**	1	49	0.410
**2**	1	49	0.212
	2	47	0.387
**3**	1	49	0.346
**4**	1	48	0.234
**5**	1	50	0.285
**6**	1	50	0.249
	2	50	0.125
**7**	1	48	0.205
	2	50	0.153
**Sum**		**490**	
**Mean**			**0.261**

Supplementary Table 5 | Statistics of the patches used for determining P_o during the pacemaker depolarization for mHCN2 channels. AP frequency 2.77 Hz.

mHCN2 - AP frequency 2.77 Hz			
**Patch No.**	**Measurement No.**	**# Traces**	**Po**
**1**	1	44	0.027
	2	44	0.086
**2**	4	99	0.181
	5	95	0.187
	6	46	0.196
**3**	7	47	0.282
	8	46	0.212
**4**	9	75	0.437
**5**	10	42	0.255
	11	49	0.258
	12	40	0.213
**Sum**		**627**	
**Mean**			**0.212**

Supplementary Table 6 | Statistics of the patches used for determining P_o during the pacemaker depolarization for mHCN4V_E channels. AP frequency 2.77 Hz.

mHCN4V_E - AP frequency 2.77 Hz			
**Patch No.**	**Measurement No.**	**# Traces**	**Po**
**1**	1	20	0.056
	2	101	0.115
	3	45	0.090
**2**	1	101	0.150
	2	50	0.091
**3**	1	40	0.140
**4**	1	97	0.304
**5**	1	101	0.556
	2	47	0.278
	3	50	0.274
**6**	1	111	0.470
	2	20	0.052
**Sum**		**783**	
**Mean**			**0.215**

Supplementary Table 7 | Statistics of the patches used for determining P_o during the pacemaker depolarization for mHCN1 channels. AP frequency 2.77 Hz.

mHCN1 - AP frequency 2.77 Hz			
**Patch No.**	**Measurement No.**	**# Traces**	**Po**
**1**	1	41	0.047
**2**	1	49	0.109
**3**	1	117	0.116
**4**	1	47	0.170
	2	42	0.212
**5**	1	48	0.259
	2	49	0.315
**6**	1	47	0.125
	2	50	0.116
**7**	1	40	0.088
	2	49	0.090
**Sum**		**579**	
**Mean**			**0.150**
