# Supplementary material for: HCN1 is a primary HCN Pacemaker Channel in Neurons
Source: Nat Commun. 2026 Apr 23;17:3745. doi: 10.1038/s41467-026-72257-3 (PMC13106851; doi:10.1038/s41467-026-72257-3)
Supplement: Supplementary file 2 — Reporting Summary [file 41467_2026_72257_MOESM2_ESM.pdf]

Corresponding author(s): Klaus Benndorf
Last updated by author(s): Mar 26, 2026

Reporting Summary

Nature Portfolio wishes to improve the reproducibility of the work that we publish. This form provides structure for consistency and transparency in reporting. For further information on Nature Portfolio policies, see our Editorial Policies and the Editorial Policy Checklist.

Statistics

For all statistical analyses, confirm that the following items are present in the figure legend, table legend, main text, or Methods section.

n/a	Confirmed	
☐	☒	The exact sample size (*n*) for each experimental group/condition, given as a discrete number and unit of measurement
☐	☒	A statement on whether measurements were taken from distinct samples or whether the same sample was measured repeatedly
☒	☐	The statistical test(s) used AND whether they are one- or two-sided<br>*Only common tests should be described solely by name; describe more complex techniques in the Methods section.*
☒	☐	A description of all covariates tested
☒	☐	A description of any assumptions or corrections, such as tests of normality and adjustment for multiple comparisons
☐	☒	A full description of the statistical parameters including central tendency (e.g. means) or other basic estimates (e.g. regression coefficient) AND variation (e.g. standard deviation) or associated estimates of uncertainty (e.g. confidence intervals)
☒	☐	For null hypothesis testing, the test statistic (e.g. *F*, *t*, *r*) with confidence intervals, effect sizes, degrees of freedom and *P* value noted<br>*Give P values as exact values whenever suitable.*
☒	☐	For Bayesian analysis, information on the choice of priors and Markov chain Monte Carlo settings
☒	☐	For hierarchical and complex designs, identification of the appropriate level for tests and full reporting of outcomes
☒	☐	Estimates of effect sizes (e.g. Cohen's *d*, Pearson's *r*), indicating how they were calculated

Our web collection on statistics for biologists contains articles on many of the points above.

Software and code

Policy information about availability of computer code

Data collection	ISO3 software with Axopatch 200B amplifier; Heka Patchmaster software with LIH8+8 hardware and OC725C amplifier
Data analysis	IgorPRO 7.08, OriginPro 2016G, MS Excel 16LTSC, Matlab R2018a, Brian2 simulation environment

For manuscripts utilizing custom algorithms or software that are central to the research but not yet described in published literature, software must be made available to editors and reviewers. We strongly encourage code deposition in a community repository (e.g. GitHub). See the Nature Portfolio guidelines for submitting code & software for further information.

Data

Policy information about availability of data

All manuscripts must include a data availability statement. This statement should provide the following information, where applicable:

- Accession codes, unique identifiers, or web links for publicly available datasets
- A description of any restrictions on data availability
- For clinical datasets or third party data, please ensure that the statement adheres to our policy

All data included in the manuscript are deposited in the Open Science Framework under osf.io/7g5ch.

Research involving human participants, their data, or biological material

Policy information about studies with human participants or human data. See also policy information about sex, gender (identity/presentation), and sexual orientation and race, ethnicity and racism.

Reporting on sex and gender	The study did not involve human participants, their data, or biological material.
Reporting on race, ethnicity, or other socially relevant groupings	The study did not involve human participants, their data, or biological material.
Population characteristics	The study did not involve human participants, their data, or biological material.
Recruitment	The study did not involve human participants, their data, or biological material.
Ethics oversight	The study did not involve human participants, their data, or biological material.

Note that full information on the approval of the study protocol must also be provided in the manuscript.

Field-specific reporting

Please select the one below that is the best fit for your research. If you are not sure, read the appropriate sections before making your selection.

[x] Life sciences [ ] Behavioural & social sciences [ ] Ecological, evolutionary & environmental sciences

For a reference copy of the document with all sections, see nature.com/documents/nr-reporting-summary-flat.pdf

Life sciences study design

All studies must disclose on these points even when the disclosure is negative.

Sample size	As the described biophysical data are observable in each data-set independently, statisticaly pre-determined sample size was not performed.
Data exclusions	For analysis and evaluation, only experiments were included if the quality was sufficiently good (adequate expression level, low and stable leak, low expression of endogeneous channels, lack of obvious electrical artefacts).
Replication	All experiments were performed with oocytes deriving from at least three different frogs, injected on different days. All attemps at replication were successful.
Randomization	Randomization was not relevant to our study since there are no experimental groups.
Blinding	Blinding was not possible, as singel-channel conductances are suffitiently different to identify the construct meassured.

Reporting for specific materials, systems and methods

We require information from authors about some types of materials, experimental systems and methods used in many studies. Here, indicate whether each material, system or method listed is relevant to your study. If you are not sure if a list item applies to your research, read the appropriate section before selecting a response.

Materials & experimental systems

n/a	Involved in the study	
[x]	[ ]	Antibodies
[x]	[ ]	Eukaryotic cell lines
[x]	[ ]	Palaeontology and archaeology
[ ]	[x]	Animals and other organisms
[x]	[ ]	Clinical data
[x]	[ ]	Dual use research of concern
[x]	[ ]	Plants

Methods

n/a	Involved in the study	
[x]	[ ]	ChIP-seq
[x]	[ ]	Flow cytometry
[x]	[ ]	MRI-based neuroimaging

Animals and other research organisms

Policy information about studies involving animals; ARRIVE guidelines recommended for reporting animal research, and Sex and Gender in Research

Laboratory animals	Xenopus laevis, aprox. 3 to 4 years old

Wild animals	The study did not involve wild animals.
Reporting on sex	The study involved 30 adult females of Xenopus laevis for oocyte extraction.
Field-collected samples	The study did not involve samples collected from the field.
Ethics oversight	All experimental procedures involving Xenopus laevis were approved by the Thüringer Landesamt für Verbraucherschutz (permit number UKJ-23-005) and were conducted in accordance with institutional guidelines of Friedrich Schiller University Jena and the German Animal Welfare Act.

Note that full information on the approval of the study protocol must also be provided in the manuscript.

Plants

Seed stocks	n.a
Novel plant genotypes	n.a.
Authentication	n.a.
